# Photosynthetic modulation during the diurnal cycle in a unicellular diazotrophic cyanobacterium grown under nitrogen-replete and nitrogen-fixing conditions

**DOI:** 10.1038/s41598-022-21829-6

**Published:** 2022-11-07

**Authors:** Michelle Liberton, Sandeep Biswas, Himadri B. Pakrasi

**Affiliations:** Department of Biology, Washington University, St. Louis, MO 63130 USA

**Keywords:** Circadian rhythms, Microbiology, Photosystem II

## Abstract

Cyanobacteria are the only oxygenic photosynthetic organisms that can fix nitrogen. In diazotrophic cyanobacteria, the regulation of photosynthesis during the diurnal cycle is hypothesized to be linked with nitrogen fixation and involve the D1 protein isoform PsbA4. The amount of bioavailable nitrogen has a major impact on productivity in aqueous environments. In contrast to low- or nitrogen-fixing (−N) conditions, little data on photosynthetic regulation under nitrogen-replete (+ N) conditions are available. We compared the regulation of photosynthesis under −N and + N conditions during the diurnal cycle in wild type and a *psbA4* deletion strain of the unicellular diazotrophic cyanobacterium *Cyanothece* sp. ATCC 51142. We observed common changes to light harvesting and photosynthetic electron transport during the dark in + N and −N conditions and found that these modifications occur in both diazotrophic and non-diazotrophic cyanobacteria. Nitrogen availability increased PSII titer when cells transitioned from dark to light and promoted growth. Under −N conditions, deletion of PsbA4 modified charge recombination in dark and regulation of PSII titer during dark to light transition. We conclude that darkness impacts the acceptor-side modifications to PSII and photosynthetic electron transport in cyanobacteria independently of the nitrogen-fixing status and the presence of PsbA4.

## Introduction

Cyanobacteria are oxygenic photosynthetic microbes with the ability to harvest light energy and convert it into chemical energy to power physiological processes. Cyanobacteria are a diverse group of organisms that are found globally in nearly all habitats, where they are major contributors to primary productivity. In natural environments, cyanobacterial cells encounter a wide variety of growth conditions that can include extreme fluctuations in nutrient availability. Nitrogen is abundant in the atmosphere, but bioavailable nitrogen requires the fixation of N_2_ gas to ammonia. The availability of nitrogen is the limiting factor in many aquatic environments.

Many strains of cyanobacteria are capable of nitrogen fixation, a process that is incompatible with photosynthesis due to the sensitivity of the nitrogenase enzyme to oxygen^1,2^. Diazotrophic cyanobacteria have developed strategies to separate these two processes: filamentous forms of cyanobacteria have evolved specialized cells called heterocysts to achieve spatial segregation of the two antagonistic processes. In these strains, nitrogen fixation occurs in the heterocysts while photosynthesis occurs in neighboring vegetative cells^3^. In contrast, unicellular diazotrophic cyanobacteria like *Cyanothece* sp. ATCC 51142 (hereafter *Cyanothece* 51142) temporally separate photosynthesis and nitrogen fixation by performing photosynthesis during the light period and nitrogen fixation during the dark as part of a complex diurnal cycle^4^. Photogenerated assimilatory power in the form of ATP and NADPH generated from the oxidation of water can be used to drive the carbon reduction required for important cellular processes including nitrogen fixation.

When growing under nitrogen-fixing conditions, *Cyanothece* 51142 cells accumulate and subsequently degrade storage inclusion bodies as part of the diurnal cycling of metabolic processes^5^. Furthermore, the nitrogenase complex is generated each day and functions during the dark period, and is then degraded late in the dark period, so that no nitrogenase proteins are present during the light period when photosynthesis occurs^6^. Multiple studies have described the transcriptome, proteome, cellular ultrastructure, and physiology of the cells under the 12 h:12 h, light:dark, nitrogen-fixing conditions^7–10^. Most studies have focused on this daily alternation of photosynthesis and nitrogen fixation when bioavailable nitrogen is scarce and nitrogen fixation is required. In contrast, when nitrogen is abundant and nitrogen fixation is not necessary, the cells do not need to produce the nitrogenase complex or accumulate carbohydrate bodies and cyanophycin granules as storage compartments^4^. Compared to nitrogen-fixing conditions, surprisingly little is known about the physiology of the cells under nitrogen-replete conditions when grown in alternating light–dark conditions, which may be a common situation in diverse natural environments for such unicellular diazotrophic cyanobacteria^11^.

Previous studies using *Cyanothece* 51142 and *Crocosphaera watsonii* WH8501 have shown that the photosynthetic apparatus is modulated under nitrogen-fixing conditions during the light:dark cycle to downregulate PSII functional activity in the dark^12,13^. The *Cyanothece* 51142 genome contains five *psbA* genes encoding different versions of the photosystem II core protein D1^14–16^. The *psbA4* gene encodes the sentinel or rogue D1 (sD1 or rD1) version that is present in many nitrogen-fixing and non-diazotrophic strains^12,17^. Transcriptomic analyses showed that the *psbA4* expression levels were highest in the dark period, in contrast to the other *psbA* genes that were preferentially expressed during the light time points^8,17^. Owing to this unique expression pattern of the *psbA4* gene, it was proposed that the PsbA4 protein is involved in this functional downregulation of PSII in the dark. In fact, expression of the PsbA4 in a *Synechocystis* sp. PCC 6803 (hereafter *Synechocystis* 6803) strain lacking all three endogenous versions of the D1 protein resulted in a strain incapable of performing photosynthesis^17^. However, the role of this protein in its native host has not been evaluated. The ability to genetically manipulate *Cyanothece* 51142^18^ now makes it possible to directly study the role of PsbA4. Toward this goal, we engineered a *Cyanothece* 51142 strain in which the *psbA4* gene is deleted. We focused on the D10 and L2 time points during the 12 h:12 h, light:dark cycle (10 h into the dark period and 2 h into the light period) to capture the transition from dark to light, time points that complement previous work that focused on the light to dark transition^17^.

In this study, we found that in *Cyanothece* 51142, PSII is functionally active in dark at the D10 time point. We also found that photosynthetic regulation is independent of nitrogen availability and PsbA4. Our results open avenues to redefine and rediscover photosynthetic regulation under nitrogen stress and role of PsbA4 in *Cyanothece* 51142.

## Results

### Growth and pigment content

**Figure 1:**
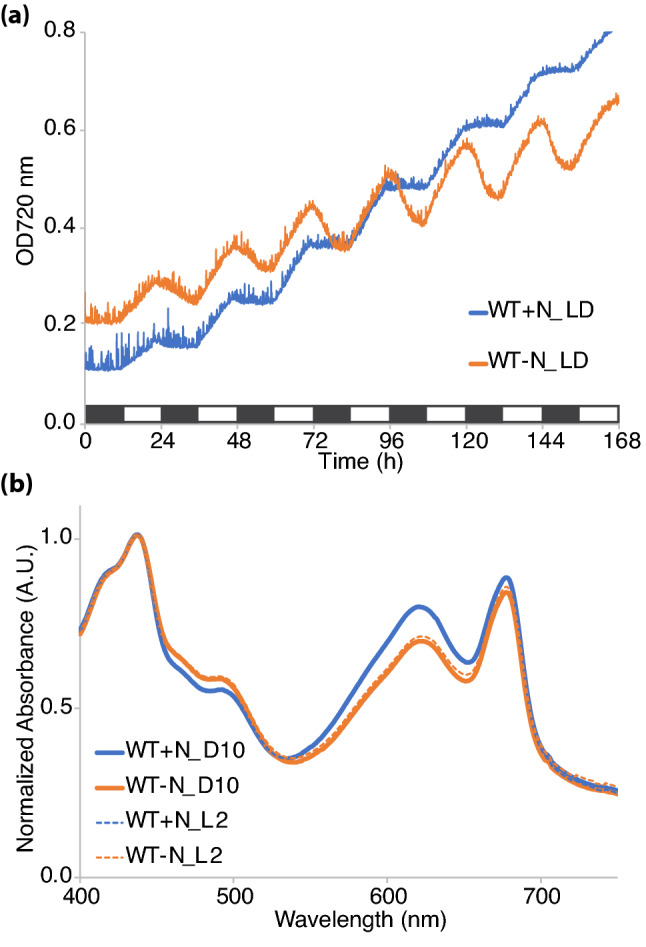
Growth and absorbance of WT *Cyanothece* sp. ATCC 51142 cultures. (**a**) Growth was measured for 7 days under 12:12 h, light:dark conditions in ASP2 + N (blue) or ASP2 − N (orange) media. Traces shown are the average of four independent growth experiments. Bar shows alternating 12 h light:dark periods. (**b**) Representative whole-cell absorption spectra collected at the D10 (solid lines) and L2 (dashed lines) time points from cultures grown in 12 h LD in + N or − N media. Data are normalized to the absorbance at 440 nm.

Photoautotrophic growth of *Cyanothece* 51142 WT cells was examined under a 12:12 h, light:dark cycle in both nitrogen replete (+ N) and nitrogen deficient (− N) ASP2 media with air bubbling at 30 °C under 100 µmol photons m^−2^ s^−1^ (Fig. 1a). At the end of the experiment, growth in ASP2 + N was higher compared to ASP2 − N as measured by the optical density at 720 nm (OD720). Under both + N and − N conditions, the cultures displayed a diurnal cycling of the OD720, with growth occurring during the light period. In the + N culture, growth occurred during the 12 h light phase, while the 12 h dark phase appeared as a plateau in the OD720. In comparison, in ASP2 − N growth occurred during the light phase but the 12 h dark phase showed a decrease in the OD720. This decrease in OD720 observed during the dark phase of growth is consistent with degradation of the polysaccharide storage bodies that are accumulated during the light and then mobilized to meet cellular energy requirements during the dark period when the cells are grown under N_2_-fixing conditions^19^.

Our measurements were collected at the D10 and L2 time points to capture the transition from dark to light. Absorption spectra were normalized to the blue absorption maxima of chlorophyll *a* (Chl*a*) at 440 nm and measured under these same + N and − N growth conditions and at the D10 and L2 time points (Fig. 1b). We observed a decrease in the 625 nm peak corresponding to phycobilin in the − N cultures compared to the + N cultures, which is consistent with a slight degradation of the phycobilisome antenna complexes. Phycobilisomes are large protein complexes that can be mobilized to make up for cellular nitrogen deficiency. The phycobilin content did not change between the D10 and L2 time points in either + N or − N conditions. The 680 nm peak corresponding to the red absorption maxima of Chl*a* showed only minor variation between + N and − N cultures. An increase in the carotenoid absorption (490 nm) was also evident under − N conditions (Fig. 1b). With the suggested role of carotenoid pigments in photoprotection and PSII assembly in cyanobacteria^20–23^, the increase in carotenoid absorption is suggestive of the vulnerability of photosystem II to damage under − N conditions.

### Low temperature fluorescence emission

Fluorescence emission measured at 77 K can provide information about PSI and PSII stoichiometry, light harvesting, and energy transfer that is not apparent from cells examined at room temperature. Depending on the excitation wavelength, fluorescence is emitted from either Chl*a* associating with PSII and PSI or from the phycobilisome antenna. Upon excitation at 435 nm (selective excitation of Chl*a*), fluorescence emission at 77 K has three major peaks: F683, F694, and F720^24^ corresponding to the PSII proximal antenna proteins CP43 and CP47, and PSI, respectively. Selective excitation of phycobilisomes is achieved by exciting complexes in the range of 550 nm to 670 nm since Chl*a* and carotenoids have a low extinction coefficient in this range^25^. Preferential excitation of phycobilisomes at 77 K shows the excitonic coupling of the phycobilisome antenna with the photosynthetic complexes^26^. Fluorescence emission with excitation at 580 nm shows the F683, F694, and F720 peaks along with two extra peaks corresponding to phycocyanin and allophycocyanin at 650 nm and 665 nm, respectively. An increase in the peak magnitude is generally accepted to indicate excitonic decoupling between the phycobilisome antenna complex and PSI or PSII.

**Figure 2:**
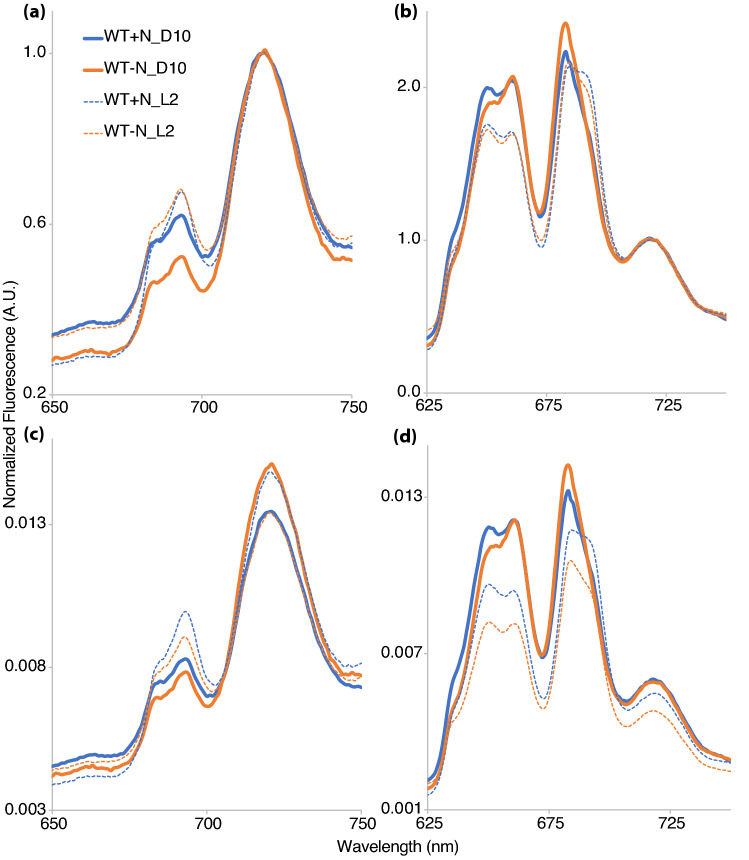
Fluorescence emission spectra at 77 K collected from WT *Cyanothece* sp. ATCC 51142 cultures. Spectra were collected at D10 (solid lines) and L2 (dashed lines) time points from cultures grown under 12:12 h, light:dark conditions in ASP2 + N (blue) or ASP2 − N (orange) media. Excitation of Chl*a* at 435 nm (**a**,**c**) and phycobilin at 580 nm (**b**,**d**). Traces shown are the average of two independent biological replicates. Traces in (**a**,**b**) were normalized to PSI fluorescence at 720 nm and (**c**,**d**) were normalized to the area under the curve.

For these measurements, cells were grown in flasks in ambient air at 30 °C under 100 µmol photons m^−2^ s^−1^ in + N or − N media and measured at D10 and L2 time points (Fig. 2). Our results showed a decline in PSII relative to PSI after dark exposure at time point D10 in N_2_-fixing conditions (− N). Upon excitation of Chl*a* at 435 nm (Fig. 2a), the 683 nm peak (corresponding to unassembled CP47 and CP43) at the D10 time point showed a decline of about 20% when compared to the L2 time point. A larger decline of approximately 22% can be observed in the 694 nm peak (corresponding to CP47 and functionally assembled PSII) when compared to the L2 time point. In comparison, cells grown under + N showed only a decline in the 694 nm peak at D10 compared to L2 (Fig. 2a).

With excitation of phycobilisomes at 580 nm (Fig. 2b), the D10 time point showed an increase in the fluorescence emission at 650 nm (allophycocyanin) and 665 nm (phycocyanin) when compared to the light time point for both + N and − N. This increase is consistent with an excitonic disconnect between the phycobilisome antenna and PSII at the dark time point. Furthermore, there was a change in the ratio of emissions at 650 nm and 665 nm with the emission becoming dominant for 665 nm at D10.

A stark observation is the change in the emissions for 683 nm and 694 nm at the D10 time point (Fig. 2b). The 683 nm peak, emanating from the terminal emitter and unassembled CP43 or CP47 complexes, is more dominant at the D10 time point. By the L2 time point, emission at 683 nm has declined while emission at 694 nm has increased to a nearly equal level, indicating an increase in the association of phycobilisome antenna with assembled PSII at L2.

Normalizing to the 720 nm emission peak as in Fig. 2a,b does not provide information on the changes in PSI under tested conditions, so we further normalized the 77 K data by the area under the curve to look at stoichiometric changes between PSII and PSI (Fig. 2c,d). The 435 nm excitation data (Fig. 2c) shows the stoichiometric changes to PSII and PSI to be opposite under + N and − N conditions. Upon transitioning from dark to light in the + N condition, we observed an increase in the PSI emission. Under − N conditions, however, the PSI emission decreased at L2 when compared to D10. The PSII emission peaks increased under light in both + N and − N conditions. The 580 nm excitation data normalized by the area under the curve shows an increase in the PSI emission peak at D10 (Fig. 2d). Since 580 nm excitation indicates the excitonic coupling of PSII and PSI with phycobilisome antenna, we propose that the increase in PSI emissions on excitation at 580 nm results from the state-transition in dark.

### Photosystem II content, assembly, and activity

**Figure 3:**
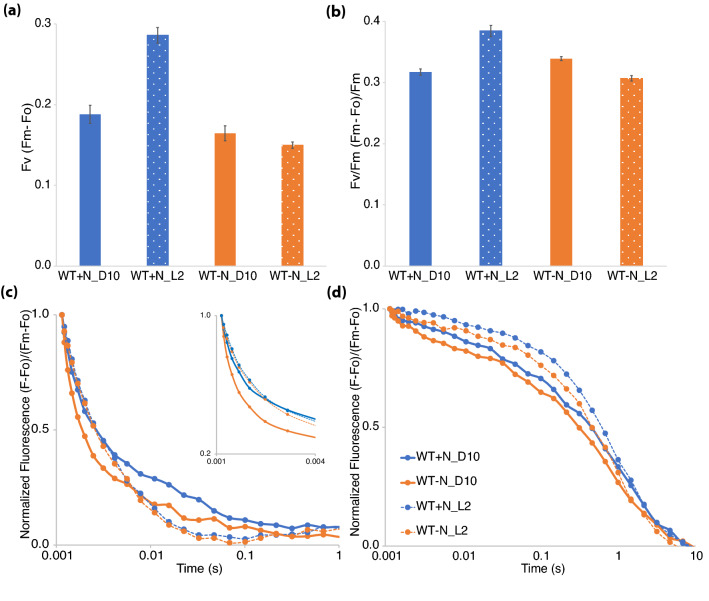
Photosystem II quantification and activity measured by following Q_A_^−^ reoxidation kinetics in WT *Cyanothece* sp. ATCC 51142. Data were collected from cultures grown under 12:12 h, light:dark conditions in + N (blue) or − N (orange) media. (**a**) Fv and (**b**) Fv/Fm measurements. Fo and Fm values were measured by exposing cells treated with 40 µM DCMU. Error bars indicate standard error of the mean from three independent biological replicates. (**c**,**d**) Q_A_^−^ reoxidation kinetics to examine acceptor-side events at D10 (solid lines) and L2 (dotted lines) time points were taken by exposing dark-adapted cells to a single blue actinic flash and blue measuring flashes at regular intervals during the measurement in the absence (**c**) and presence (**d**) of DCMU. Only 1 s of measurement is presented in (**c**) to emphasize the fast and intermediate relaxation phases. To further display the initial fast phase, the first 4 ms of measurement is enlarged in the inset in (**c**). Total 2 ml of culture adjusted to 3 µg ml^−1^ Chl*a* concentration was used for each measurement. Data are the average of three independent biological replicates.

When dark-adapted cyanobacterial cells are exposed to a strong actinic light, the measured difference between the basal fluorescence level (Fo) and the maximum fluorescence (Fm) in the presence of DCMU reports on the number of active PSII centers in the sample. DCMU is a plastoquinone analog that prevents electron flow from Q_A_ to Q_B_ in PSII. This measure, Fv (Fm-Fo), was higher at L2 compared to D10 when cells were grown in + N (Fig. 3a). When cells were grown in − N, Fv was slightly lower at L2 compared to D10. This suggests that the number of active PSII centers was higher at the L2 time point when cells were grown in + N conditions, and that there was a small decline in the number of active PSII centers between D10 and L2 when cells were grown in − N conditions. Photosynthetic efficiency (Fv/Fm) was higher at L2 for + N conditions but lower at L2 compared to D10 for − N conditions (Fig. 3b).

Q_A_^−^ reoxidation kinetics was used to study the acceptor-side events in PSII (Fig. 3c). Curiously, the fluorescence relaxation was faster at D10 for the first 4 ms post-exposure to a single saturating flash. Like Sicora et al., 2018, we also observed a delay in the relaxation between the 10 ms to 1 s of the fluorescence relaxation curve at the D10 time point. This effect was more severe in the + N condition compared to − N. The initial fast fluorescence relaxation could be due to the plastoquinone bound at the Q_B_ site being in an oxidized state in the dark, while the delay in the fluorescence relaxation between 10 ms and 1 s could be indicative of delayed plastoquinone exchange with the plastoquinone pool^27^. Cells at the D10 time point also showed a faster fluorescence quenching with DCMU in both + N and − N (Fig. 3d) indicating a faster charge recombination between Q_A_^−^ and the S2 state of Mn. However, the large-scale disassembly of PSII, as has been postulated in previous studies involving *Cyanothece* 51142, could be ruled out (see discussion section for further details).

To determine whether the pattern of fluorescence relaxation was specific to *Cyanothece* 51142 or whether it also occurred in nondiazotrophic cyanobacteria, we measured Q_A_^−^ reoxidation kinetics in *Synechocystis* 6803 (Supplementary Fig. S1), a unicellular strain that is not able to fix nitrogen. In measurements taken at L2 and D10 time points from *Synechocystis* 6803 cells growing in 12:12 h, light:dark conditions in BG11 media, we observed the same initial fast kinetics followed by a delayed fluorescence relaxation (Supplementary Fig. S1). This suggests that nitrogen fixing capability does not impact the pattern of fluorescence relaxation under dark and light, which could be a more general feature of PSII encountering darkness.

### Photosystem I redox kinetics during the diurnal cycle

**Figure 4:**
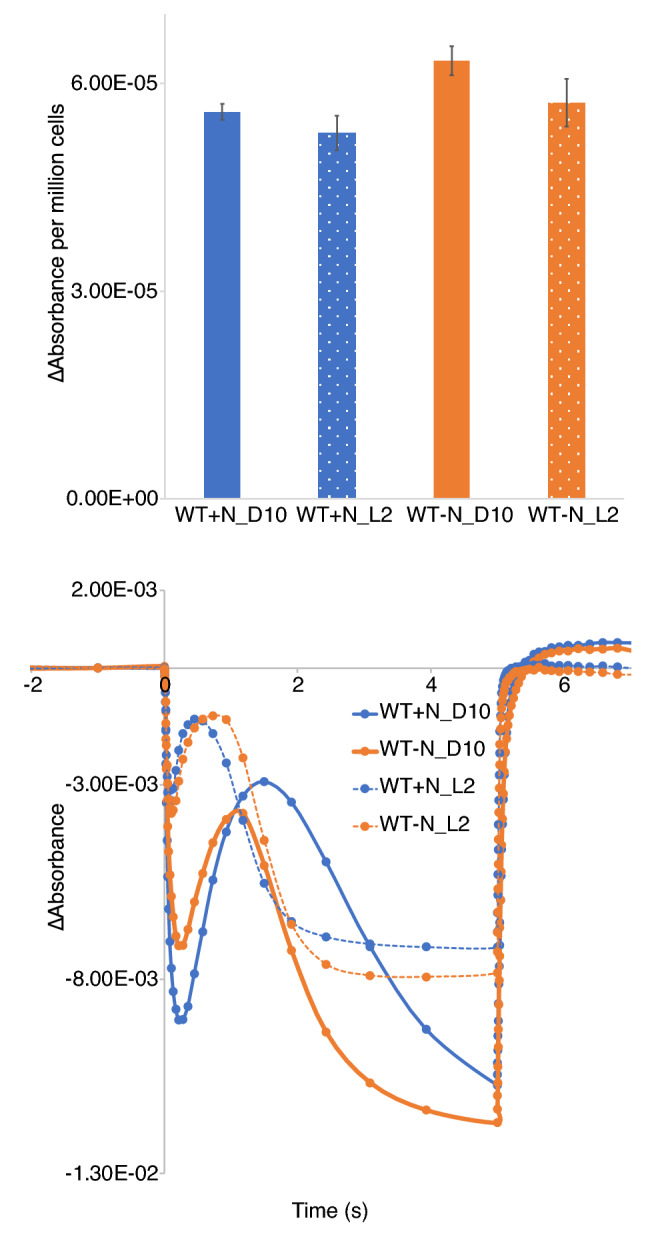
Photosystem I quantification and redox kinetics in WT *Cyanothece* sp. ATCC 51142. Data were collected from cultures grown under 12:12 h, light:dark conditions in + N (blue) or − N (orange) media. (**a**) Absorbance change at 705 nm in dark-adapted cells exposed to actinic light at 630 nm for 5 s in cells adjusted to 3 µg ml^−1^ Chl*a* in the presence of 40 μM DCMU and 1 μM DBMIB. Data are presented as absorbance change per million cells for D10 and L2 time points. Error bars indicate standard error of the mean from two independent biological replicates. (**b**) P700 redox kinetics collected from cells at D10 (solid lines) and L2 (dashed lines) time points. Data shown are the average of two independent biological repeats.

The status of PSI function was examined by following the P700 (PSI reaction center) redox kinetics in cells collected at D10 and L2 timepoints. The change in absorbance measured at 705 nm with the inhibitors DCMU and DBMIB is representative of the number of PSI centers present in the sample. These data showed that the − N cultures contain slightly more PSI per 10^6^ cells compared to + N cultures, but there is no significant difference between the D10 and L2 time points (Fig. 4a).

The redox kinetics for P700 showed some differences between + N and − N as well as between L2 and D10 (Fig. 4b). One of the major changes between the L2 and D10 time points was the enhanced oxidation of P700 at D10 upon being exposed to light (Fig. 4b). Another aspect of the P700 oxidation between the dark and light time points was the delay in P700 reduction on exposure to actinic light. Since linear electron transport is the major contributor of electrons leading to the reduction of P700, the data presented suggests a reduced influx of electrons from PSII at D10. The dark reduction kinetics of P700^+^ was also very different between the dark and light time points. The recovery of P700 was faster for the L2 samples when compared to D10. Recovery half-times for D10 samples between the treatments ranged from ~ 50 ms to ~ 70 ms, while the half-times recorded for L2 were ~ 20 ms. The data and recorded half times would suggest that the changes in the linear electron transport are not due to the nitrogen-fixing activity of the cells but rather result from growth in light vs dark. Enhanced oxidation of P700 on exposure to light at D10 could also be a result of state transition. Coupling of phycobilisome antenna with PSI under dark could be responsible for channelizing energy directly to PSI that would result in increased oxidation of P700 under dark. These data further suggest a large-scale decline in PSII activity in the dark that occurs in both + N and − N growth conditions.

### Analysis of the ∆*psbA4* strain

A *psbA4* deletion strain was constructed in *Cyanothece* 51142 by replacing the *psbA4* coding region with a gene encoding kanamycin resistance, and PCR and DNA sequencing was used to verify successful deletion of *psbA4* (Supplementary Fig. S2). Western blotting was used to confirm that the PsbA4 protein was present in the WT but not in the ∆*psbA4* strain. We furthermore showed the presence of PsbA4 at the D10 time point but not at the L2 time point in the WT strain (Supplementary Fig. S2).

**Figure 5:**
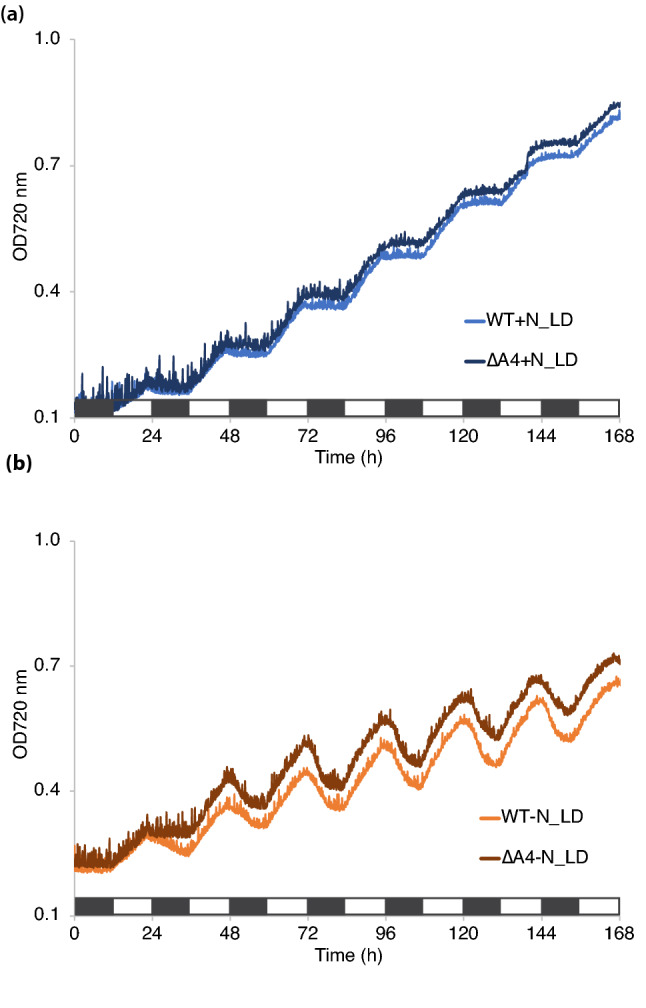
Growth of WT and ∆*psbA4* strains of *Cyanothece* sp. ATCC 51142. Growth was measured for 7 days under 12:12 h, light:dark conditions in ASP2 + N (blue) or ASP2 − N (orange) media. Traces shown are the average of four independent growth experiments. Bar shows alternating 12 h light:dark periods.

We compared growth of the WT and mutant strains under different conditions. In + N conditions with air bubbling at 30 °C under 100 µmol photons m^−2^ s^−1^, both strains showed similar growth (Fig. 5a). When grown under the same conditions but without added nitrogen (− N), our results showed slightly accelerated growth for the mutant compared to WT (Fig. 5b). The pigment composition of WT compared to the ∆*psbA4* strain was similar in + N and − N, and at the different time points (Supplementary Fig. S3).

Under + N conditions, fluorescence emission measured at 77 K remained similar for the WT and *∆psbA4* strains (Supplementary Fig. S4). Under −N conditions, fluorescence emission profiles with excitation at 435 nm (Supplementary Fig. S4) showed an increase in the ratio of F694 to F683 of nearly 22% in the *∆psbA4* strain compared to a 16% increase for the WT strain for the L2 time point, indicating a larger increase in functional PSII accumulation in the mutant. A similar trend was observed for 580 nm excitation (Supplementary Fig. S4). PSI redox kinetics (Supplementary Fig. S5) of the ∆*psbA4* strain did not reveal any major differences between the WT and mutant strains.

**Figure 6:**
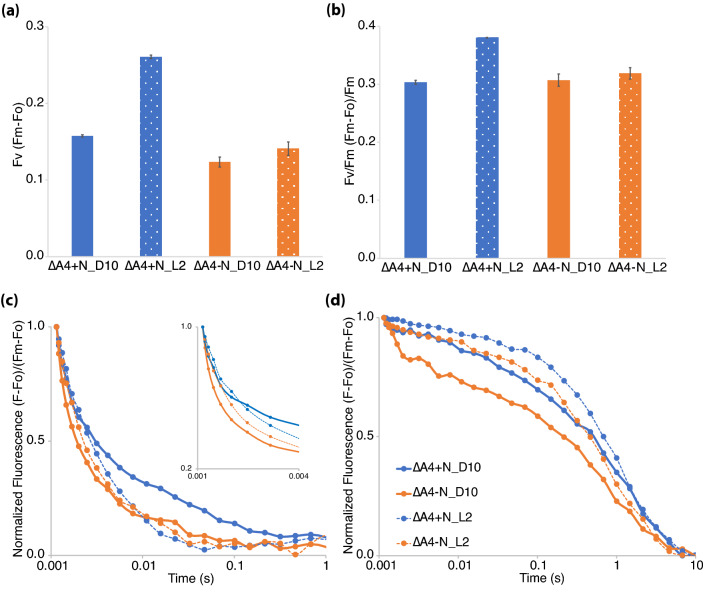
Photosystem II quantification and activity measured by following Q_A_^−^ reoxidation kinetics in the ∆*psbA4* strain of *Cyanothece* sp. ATCC 51142. Data were collected from ∆*psbA4* cultures grown under 12:12 h, light:dark conditions in + N (blue) or − N (orange) media. Fv (**a**) and Fv/Fm (**b**) measurements. Fo and Fm values were measured by exposing cells treated with 40 µM DCMU. Error bars indicate standard error of the mean from three independent biological replicates. (**c**,**d**) Q_A_^−^ reoxidation kinetics to examine acceptor-side events at D10 (solid lines) and L2 (dotted lines) time points were taken by exposing dark-adapted cells to a single blue actinic flash and blue measuring flashes at regular intervals during the measurement in the absence (**c**) and presence (**d**) of DCMU. Only 1 s of measurement is presented in (**c**) to emphasize the fast relaxation phase. To further display the initial fast phase, the first 4 ms of measurement is enlarged in the inset in (**c**). Total 2 ml of culture adjusted to 3 µg ml^−1^ Chl*a* concentration was used. Data are the average of three independent biological replicates.

Since *psbA4* is an isoform of the commonly expressed *psbA1* and *psbA5* copies of the D1 protein, we further focused on the impacts of removing *psbA4* on amount of active PSII and PSII-associated electron transfer events. Curiously, unlike the WT strain (Fig. 3a-b), Fv and Fv/Fm showed a slight increase in Δ*psbA4* cells grown under  − N condition during transitions from dark to light (Fig. 6a,b). For cells grown under + N conditions, both Fv and Fv/Fm followed the same increasing trend as was observed for the WT strain. The acceptor-side events (Fig. 6c) in the Δ*psbA4* strain were very similar to what was observed for WT. However, the charge recombination kinetics at D10 was much faster in Δ*psbA4* when compared to wild type (Fig. 3d and Fig. 6d) for cells grown under − N conditions. In general, the charge recombination was observed to be faster at D10 than at L2 for both + N and − N conditions.

## Discussion

We have compared photosynthetic regulation under nitrogen-replete and N_2_-fixing conditions in the unicellular diazotrophic cyanobacterium *Cyanothece* 51142 grown under a diurnal cycle. Regulation of the photosynthetic machinery under N_2_-fixing conditions has been examined in unicellular diazotrophic cyanobacteria grown under light:dark conditions^12,13,17^. These studies have suggested that the cycling of photosynthesis and nitrogen fixation during light:dark growth and the observed decrease in photosynthetic activity in the dark function to promote nitrogen fixation. However, a comparison of + N and − N conditions has been lacking until now. Our finding of similar modulation of photosynthetic activity between + N and − N conditions suggests to us that the observed photosynthetic modulation could be a general feature among oxygenic cyanobacteria. In the following paragraphs, we discuss the possible causes for the modifications to photosynthetic electron transport in the dark. We also discuss the possible changes nitrogen availability may have on regulating the amount of active PSII and PSI.

Nitrogen fixation is an energy-intensive process that uses the photosynthetically derived assimilatory power in the form of ATP. When nitrogen is abundant and N_2_-fixation is not required, growth as measured by OD720 is higher in *Cyanothece* 51142 cultures compared to nitrogen-fixing conditions (Fig. 1a). In cultures grown under a 12:12 h, light:dark cycle, cell growth occurred during the light period, as seen by an increase in OD720 during the light in both + N and − N cultures. In contrast, cultures displayed a sharp decline in OD720 during the dark phase when cells are fixing nitrogen (Fig. 1a), likely due to the breakdown of polysaccharide bodies to fuel N_2_-fixing activity^5^. Thus, the growth data reflect the combination of the accumulation and later mobilization of inclusion bodies and the increase in cell number during the diurnal cycle. Cyanobacterial cells degrade phycobilisomes as an immediate source of nitrogen for sustaining growth^28^, which was evident from the lowered phycobilin content seen under − N conditions (Fig. 1b).

Low-temperature (77 K) fluorescence emission profiles were similar between WT *Cyanothece* 51142 cells grown in + N and − N (Fig. 2). Excitation at 435 nm (Chl*a*) and 580 nm (phycobilin) showed minor changes in fluorescence emission peaks between the D10 and L2 time points that were observed under both + N and − N conditions, suggesting that the modifications to the photosynthetic machinery is not dependent on the nitrogen-fixing status of the cells. However, further assays to determine active-PSII and -PSI amounts revealed interesting impacts of nitrogen availability. The amount of active PSII (Fv) of cells normalized on a Chl*a* basis remained similar between the + N and − N conditions at the D10 time point (Fig. 3a). However, during the transition of cells from dark to light, nitrogen availability impacts active PSII amounts. Under + N conditions, an increase in Fv is observed at L2 compared to D10, but in − N the cells showed a slight reduction in Fv at L2 compared to D10. This increase in Fv at the L2 time point was also observed in *Synechocystis* 6803 (Supplementary Fig. S1). Interestingly, the amount of PSI was higher under N_2_-fixing conditions compared to + N (Fig. 4a). Upon transition from dark to light, both conditions revealed a small reduction in per cell PSI content. Our results indicated that the faster growth of cells under + N conditions is not only dependent on availability of resources but also due to an immediate increase in PSII amounts when cells are exposed to light.

Studies involving *Cyanothece* 51142 and *Crocosphaera watsonii* WH8501 have suggested that the photosynthetic electron transport is downregulated to promote nitrogen fixation during dark^12,13,17^. Our observations suggest that the presence or absence of nitrogen does not play a role in regulating photosynthetic electron transport. We found that dark, regardless of nitrogen availability, seems a more probable reason for the delayed fluorescence quenching that has been observed in this and previous studies (Fig. 3c). To strengthen our inference, we compared the fluorescence quenching between *Cyanothece* 51142 and *Synechocystis* 6803 cells grown under a 12:12 h, light:dark cycle (Fig. 3c and Supplementary Fig. S1). Both cyanobacterial strains exhibited a similar delay in the fluorescence quenching for the dark time point, which was possibly due to the delay in plastoquinone exchange. In our experiments, cells at the D10 time point exhibited faster fluorescence quenching with DCMU (Fig. 3d and Supplementary Fig. S1). The accelerated charge recombination between the acceptor- and donor-side of PSII can depend on the changes in the plastoquinone redox state of the cells and charge distribution equilibrium between the two PSII-bound plastoquinones, Q_A_ and Q_B_, or on the status of cytochrome *b*559^29,30^. Cytochrome *b*559 is an integral part of oxygenic PSII^31,32^ and can exist at various redox potentials^33^. Most importantly, aerobic and anaerobic conditions also impact the redox potential of cytochrome *b*559^34^. The changes in PSII-related electron transport seen in *Cyanothece* 51142 during the dark phase could also result from state 1 to state 2 transition. Either one or more of these potential alterations may lead to the observed changes to photosynthetic electron transport in the dark and are topics for future study.

The P700-associated absorbance change in cells subjected to dark (D10) suggested a delayed influx of electrons from PSII (Fig. 4b) that could be due to state 1 to state 2 transitions, as is suggested from the increased 720 nm peak in dark in the 77 K emission spectra (Fig. 2d). The 77 K fluorescence emission upon 435 nm excitation does not show changes to the F683 and F694 emission peaks that would suggest large-scale PSII disassembly in dark (Fig. 2a,c). Furthermore, fluorescence quenching with DCMU suggests that PSII is intact in dark (Fig. 3d). Disassembly of PSII and concomitant loss of the Mn cluster results in decreased PSII activity^35^. Fluorescence quenching is abolished in cells treated with hydroxylamine, which is known to remove the Mn cluster^36^. The intact charge recombination with DCMU treatment suggests that the Mn cluster and PSII are stable.

In cyanobacterial genomes, the genes encoding for photosynthetic reaction center proteins are typically found as multiple copies, resulting in the presence of distinct D1 protein versions^14,15^. Phylogenetic analyses of D1 proteins have identified several classes of these variant forms, which are proposed to provide organisms with the ability to adapt to varying environmental conditions^15,37–39^. In *Cyanothece* 51142, PsbA4 is the most divergent of the four D1 isoforms and *psbA4* is preferentially expressed in dark in N_2_-fixing conditions^8,17^. Lack of the LDLA motif in *psbA4* suggested that C-terminal processing does not take place, preventing Mn-cluster assembly^17^. The expression pattern and unusual C-terminus suggested that PsbA4 acts as a sentinel or placeholder in PSII complexes during the dark period^17^. Proteomics data suggested low abundance of PsbA4 in the dark with a concomitant decline in PSII dimers^12,40,41^. The large stoichiometric difference between functional PSII and PsbA4-containing complexes led to the suggestion of an unidentified enzymatic function for PsbA4-containing complexes^12^.

We generated a ∆*psbA4* deletion mutant in *Cyanothece* 51142 to further clarify the role of PsbA4 in the interplay of photosynthesis and nitrogen fixation. In our study, we found several differences between WT and mutant during growth in −N conditions. The growth difference (Fig. 5b), small change in functional PSII titer (Fig. 6a and Supplementary Fig. S4) and faster charge recombination (Fig. 6d) were the most apparent differences between the two strains. A slight growth advantage under −N conditions on removing PsbA4 is consistent with the increase in PSII titer (Fig. 6a) observed during the D10 to L2 transition. The slight decrease in PSII titer for WT under −N conditions at L2 compared to D10 (Fig. 3a) would be consistent with the decline in the D1 protein levels observed during early hours of transition from dark to light noted by Sicora and colleagues^13^. The faster charge recombination (Fig. 6d) observed by the ∆*psbA4* strain at the D10 time point under −N conditions suggests that PsbA4 might be influencing the electrochemical potential of the redox cofactors associated with PSII. Measurements using isolated PSII complexes from the dark growth phase could provide insights into the mechanism behind this observation. In contrast, under + N conditions the large increase in PSII titer in both strains observed during the dark to light transition (Figs. 3a and 6a) would suggest that the regulation of PSII titer is specifically related to nitrogen-fixing activity.

Together, these results challenge the general assumption that nitrogen availability is the driving stimulus behind changes to photosynthetic electron transport. We found that the availability of light has a greater impact on the acceptor and donor side modifications to PSII. Furthermore, the regulation of photosynthetic electron transport occurs independently of PsbA4, although PsbA4 might regulate the amount of active PSII when cells are fixing nitrogen. Since nitrogenase activity is abolished under complete absence of oxygen, which might happen after prolonged dark^6^, early light hours might create a microaerobic condition to promote nitrogen fixation for a short period in natural environments^42,43^. During those early hours, PsbA4 could act to prevent active PSII repair, which would lead to a reduction in the amount of PSII as a lack of PSII-assembly subunits prevents de novo PSII assembly. Such a mechanism might be advantageous under field conditions for the growth of a diazotrophic organism. This study opens avenues to redefine and rediscover photosynthetic regulation under nitrogen stress and the role of PsbA4 in *Cyanothece* 51142.

## Methods

### Mutant construction

We constructed the ∆*psbA4* strain in *Cyanothece* 51142 by replacing the entire open reading frame of the *psbA4* gene with a kanamycin cassette. The *psbA4* upstream and downstream regions and the kanamycin resistance cassette were amplified using primers designed to have overlaps on the 3’ and 5’ ends to facilitate ligation using the Gibson assembly protocol. The plasmid pRL271 was digested with BstI and XhoI and the plasmid backbone was gel extracted. The purified PCR fragments and the plasmid backbone were assembled and verified by sequencing. The resultant plasmid was introduced into the wild type (WT) strain of *Cyanothece* 51142 cells by conjugation as previously described^18^. Segregation was verified by the absence of the *psbA4* gene by PCR using primers that amplified an internal 300 bp region of *psbA4* and by sequencing.

### Maintenance of cell culture

Mutant and WT *Cyanothece* 51142 cell lines were maintained on BG-11 agar plates at 30 °C under constant illumination of 30 μmol photons m^−2^ s^−1^ supplied by LED lighting. For the ∆*psbA4* strain, kanamycin was added at a final concentration of 10 μg ml^−1^. The plates were restreaked every 20 days on fresh agar plates.

### Liquid starter culture and cell preparation for physiological assays

Cells were collected from agar plates and resuspended in Erlenmeyer flasks in ASP2 medium containing 17.65 mmol NO_3_^−^ (ASP2 + N). The cells were grown at 30 °C under 50 μmol photons m^−2^ s^−1^ continuous LED light with shaking for 6 days. From these cultures, cells were diluted at 1/10^th^ volume into ASP2 media with or without nitrate, depending on the experiment, and grown under 50 μmol photons m^−2^ s^−1^ continuous LED light with shaking for 4–5 days. From these cultures, cells were transferred to flasks with ASP2 media either with or without nitrogen at 1/10th volume and grown for another 6 days at 30 °C under 100 μmol photons m^−2^ s^−1^ cool white fluorescent 12:12 h light:dark conditions in flasks with shaking. For physiological assays, cells were harvested by centrifugation at 5000×*g* for 5 min. The cells were washed twice with ASP2 − N medium and resuspended to 3–5 μg ml^−1^ Chl*a* in fresh ASP2 medium. Chl*a* content was measured as detailed previously^44^.

### Whole-cell absorption and photoautotrophic growth

Whole-cell absorption was measured using DW2000 (Olis, Inc., USA) in disposable plastic cuvettes with 1 cm pathlength. For photoautotrophic growth, cells were added to fresh ASP2 + N or ASP2 − N media in glass tubes to a final volume of 60 ml with the optical density at 720 nm (OD720) between 0.1 and 0.2. Growth was continuously monitored (every 5 min) for a period of 7 days using the MC1000 Multicultivator (Photon Systems Instruments, Czechia). The starting OD720 of the + N cultures was adjusted lower compared to the −N cultures to avoid oversaturation of the OD measurement during the course of the experimental period. The MC 1000 was set to maintain temperature at 30 °C and light intensity of 100 μmol photons m^−2^ s^−1^ during the growth period.

### Fluorescence-based assays at cryogenic and room temperature

Low temperature (77 K) fluorescence emission was carried out by freezing a cell suspension around glass rods. Emission spectra were recorded using a FluoroMax 2 (ISA, Inc., USA). Excitation wavelengths of either 435 nm (for excitation of Chl*a*) or 580 nm (for excitation of phycobilisome antenna) were used. Room temperature fluorescence relaxation measurements were performed using a FL200 (Photon Systems, Inc., Czechia). Cells (2 ml adjusted to 3 μg ml^−1^ Chl*a*) were dark-adapted for 5 min before measurements. The blue actinic light intensity was 100% and the actinic flash duration was 50 µs. To eliminate the actinic light effect, data were recorded 150 μs after the actinic flash. 3-(3,4-dichlorophenyl)-1,1-dimethylurea (DCMU) was added where indicated at a concentration of 40 µM. The variable fluorescence (Fv) and photosynthetic efficiency (Fv/Fm) were calculated in the presence of DCMU. Amount of active photosystem II was represented as the change in Fv between the D10 and L2 time points.

### Photosystem I quantification and redox kinetics

Photosystem I reoxidation kinetics were measured using a Joliot-type spectrophotometer (JTS) 150 (Spectrologix, USA). Cells (3 ml) were resuspended to 3 μg ml^−1^ Chl*a* and dark-adapted for 5 min prior to measurement. Actinic light (630 nm) intensity was set at 1500 μmol m^−2^ s^−1^ and a P700 cutoff filter was used to measure the photosystem I specific absorbance change. Photosystem I content was quantified by recording redox kinetics in the presence of 1 μM 2,5-dibromo-6-isopropyl-3-methyl-1,4-benzoquinone (DBMIB) and 40 μM DCMU followed by cell counting using the Cellometer Auto M10 (Nexcelom Bioscience, USA) to present the change in P700 absorbance per million cells.

### Protein isolation and western blot

Total 500 ml of *Cyanothece* 51142 cells were harvested at each timepoint by centrifugation at 5000×*g* for 5 min from cultures grown in Roux bottles under 12:12 h, light:dark conditions. Light intensity was kept at 100 μmol m^−2^ s^−1^ and the cultures were constantly bubbled with air using an aquatic air pump. After harvesting, the cells were stored at − 80 °C until further use. Cells were broken in buffer (25 mM MES, 5 mM CaCl_2_ and 10 mM MgCl_2_; pH adjusted to 6.5 with NaOH) by beating with glass beads at 0 °C to isolate total membranes. To solubilize membranes, samples were incubated at 0 °C for 30 min with β-DDM (final concentration 1%) in buffer and centrifuged at 16,000×*g* for 20 min to remove impurities. The solubilized membranes were separated on a 12.5% SDS-PAGE gel and transferred onto PVDF membrane. The anti-PsbA4 antibody was a generous gift from Dr. Joseph Komenda^12^ and was used at 1:5000 dilution.

## Supplementary Information

Supplementary Figures.

## Data Availability

The data that support the findings of this study are available from the corresponding author upon reasonable request.
