# Supplementary material for: Photosynthetic modulation during the diurnal cycle in a unicellular diazotrophic cyanobacterium grown under nitrogen-replete and nitrogen-fixing conditions
Source: Sci Rep. 2022 Nov 7;12:18939. doi: 10.1038/s41598-022-21829-6 (PMC9640542; doi:10.1038/s41598-022-21829-6)
Supplement: Supplementary file 1 — Supplementary Figures. [file 41598_2022_21829_MOESM1_ESM.pdf]

Photosynthetic modulation during the diurnal cycle in a unicellular diazotrophic cyanobacterium grown under nitrogen-replete and nitrogen-fixing conditions

Michelle Liberton, Sandeep Biswas, and Himadri B. Pakrasi

Department of Biology, Washington University, St. Louis MO 63130, USA

Supplementary Data

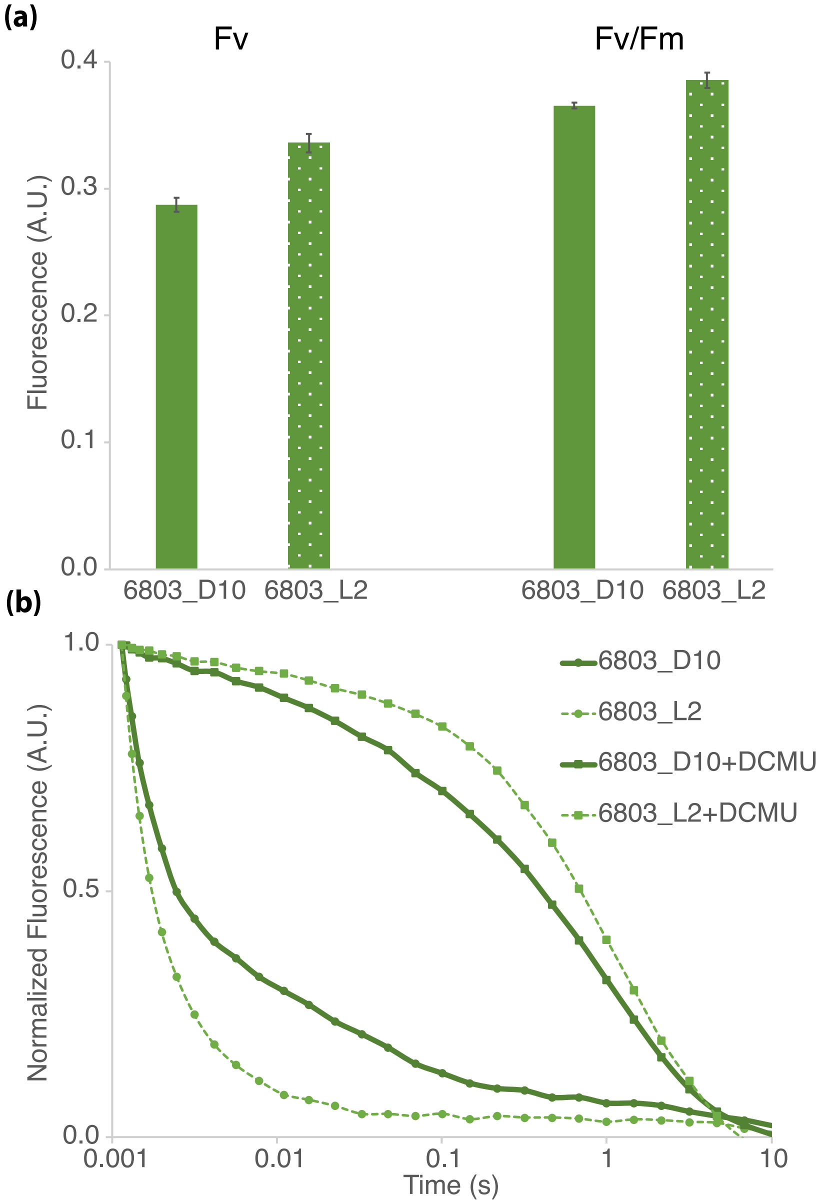

Figure S1. Photosystem II quantification and activity measured by following Q_A^- reoxidation kinetics in *Synechocystis* sp. PCC 6803. Data were collected from cultures grown under 12 : 12 h, light : dark conditions in BG11 media. (a) Fv and Fv/Fm measurements were the average of four independent biological replicates. Error bars show standard error of the mean. Fo and Fm values were measured by exposing cells treated with DCMU. (b) Q_A^- reoxidation kinetics to examine acceptor-side events at D10 (solid lines) and L2 (dotted lines) time points were taken by exposing dark-adapted cells to a single blue actinic flash and blue measuring flashes at regular intervals during 10 s of measurement in the absence or presence of DCMU. Total 2 ml of culture adjusted to 3 μg ml^{-1} Chl*a* concentration was used. Data shown are the average of four independent biological replicates.

Figure S1

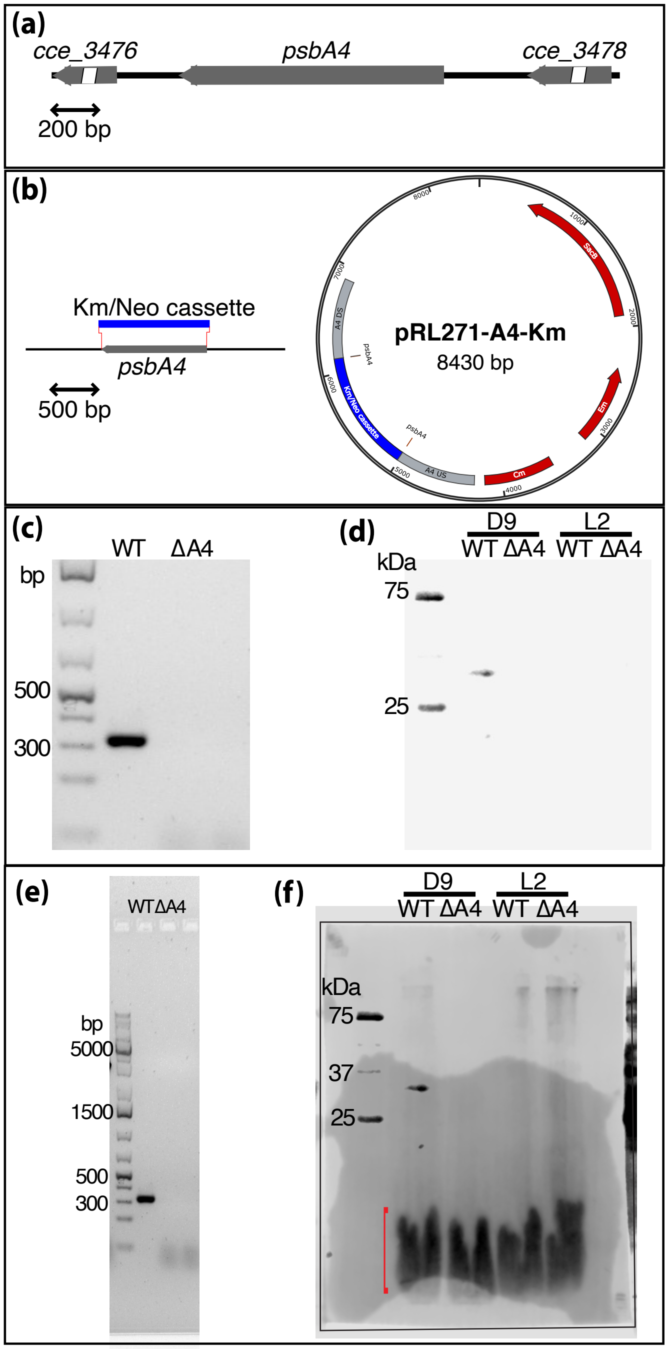

Figure S2. Construction and verification of the Δ*psbA4* strain in *Cyanothece* sp. ATCC 51142. (a) Diagram of genome organization. (b) Plasmid construction and plasmid map showing replacement of the *psbA4* gene with a kanamycin/neomycin resistance cassette. US and DS designate the upstream and downstream regions of *psbA4*. (c) Verification of the mutant strain by PCR using internal primers to show absence of the *psbA4* gene. (d) Western blotting using rD1 antibody. Gel and blot images in (c) and (d) have been cropped for clarity; (e) and (f) show full length/uncropped versions. Chlorophyll fluorescence (red bracket) is shown in (f) by the 700 channel superimposed on the chemiluminescence channel recorded on a Li-Cor ImageQuant LAS-4000 imager. The molecular weight standards used were the Thermo-Fisher GeneRuler 1 kb Plus DNA ladder (c and e) and Bio-Rad Precision Plus Dual Color Protein Standard (d and f).

Figure S2

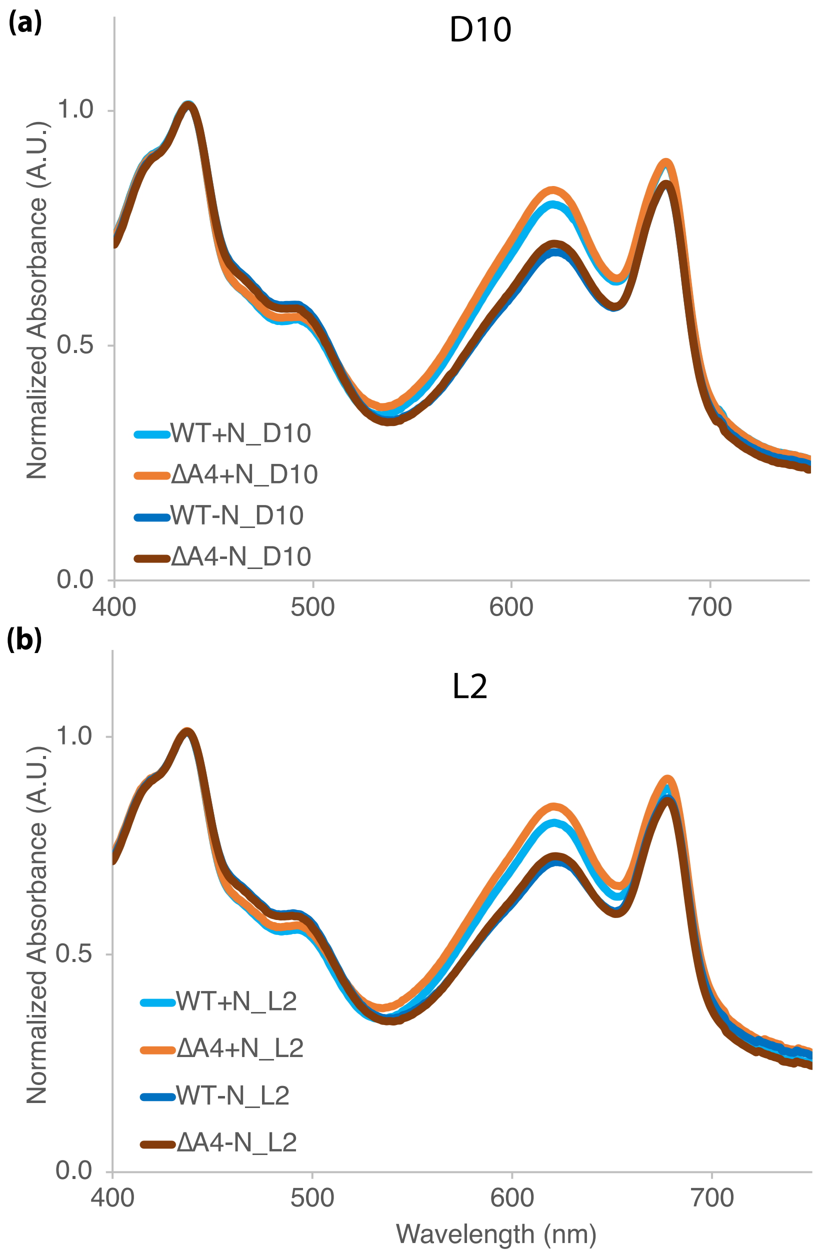

Figure S3. Absorbance of wild type and Δ*psbA4 Cyanothece* sp. ATCC 51142 strains. Representative whole-cell absorption spectra collected at the (a) D10 and (b) L2 time points from cultures grown in 12 :12 h, light : dark conditions in ASP2+N or ASP2-N media. Data are normalized to the absorbance at 440 nm.

Figure S3

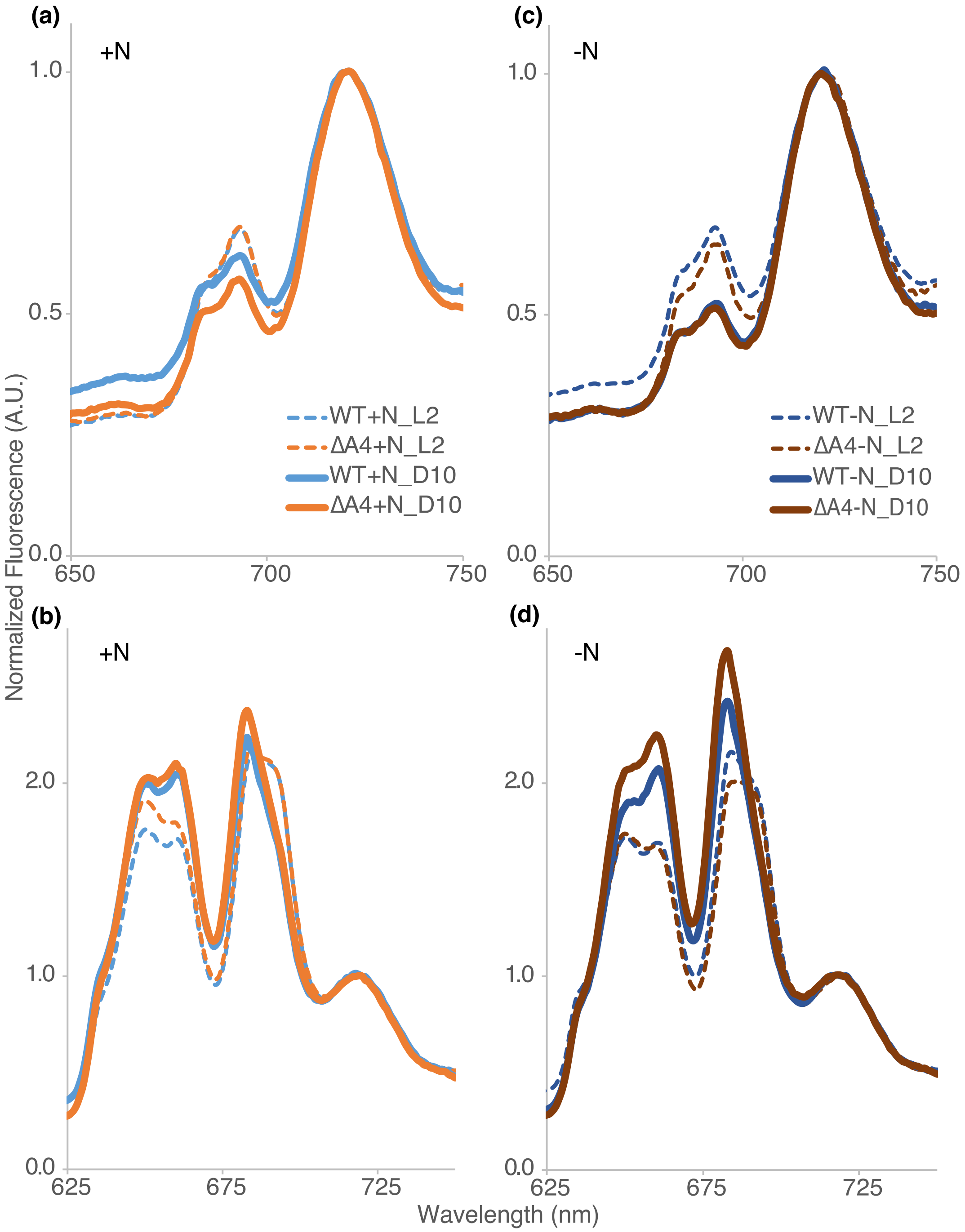

Figure S4. Fluorescence emission spectra at 77 K measured from wild type and Δ*psbA4 Cyanothece* sp. ATCC 51142 strains. Spectra were collected at D10 and L2 timepoints from cultures grown under 12 : 12 h, light : dark conditions in ASP2+N or ASP2-N media. Excitation was for chlorophyll at 435 nm (a,c) and phycobilin at 580 nm (b,d). Traces were normalized to PSI fluorescence at 720 nm. Traces shown are the average of two independent biological replicates.

Figure S4

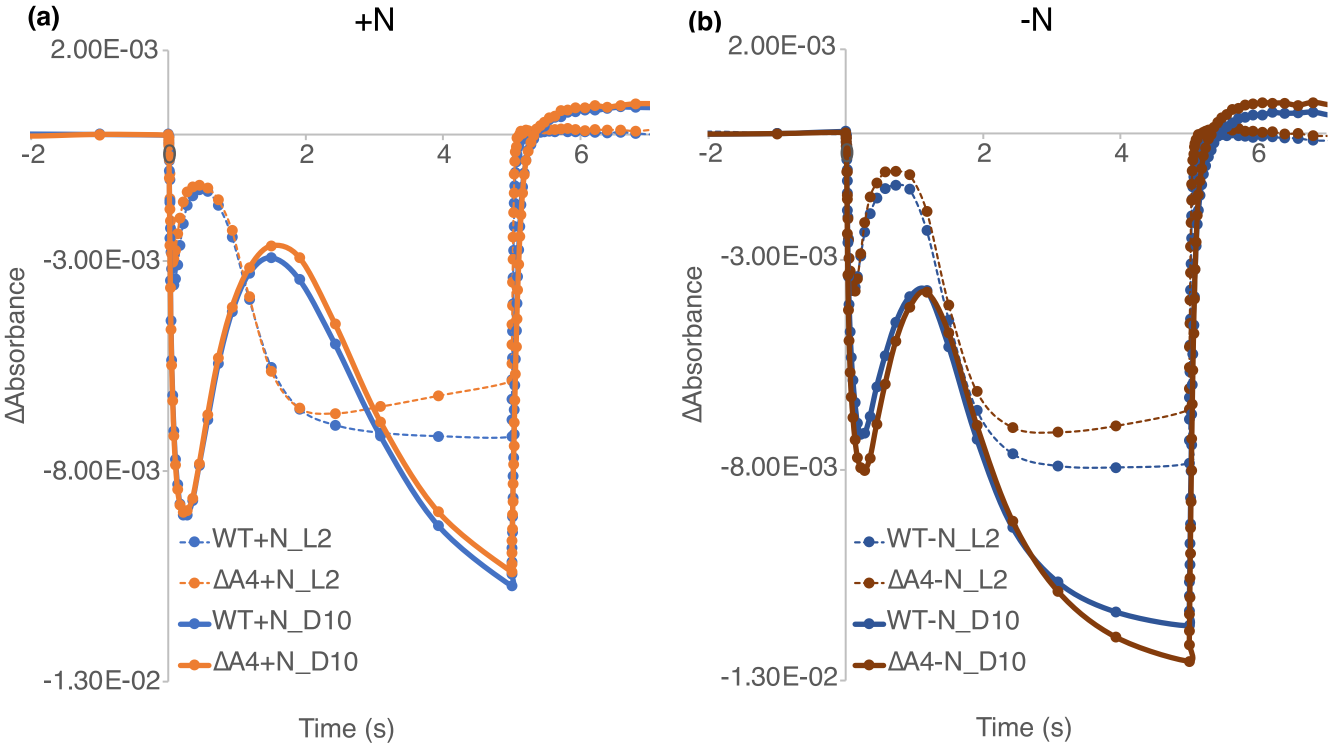

Figure S5. Photosystem I redox kinetics measured from wild type and Δ*psbA4 Cyanothece* sp. ATCC 51142 strains. Data were collected from cultures grown under 12 : 12 h, light : dark conditions in (a) ASP2+N or (b) ASP2-N media at the D10 and L2 time points. Cells were adjusted to 3 μg ml^{-1} Chl*a*. Data shown are the average of two independent biological replicates.

Figure S5
